# Natural high-avidity T-cell receptor efficiently mediates regression of cancer/testis antigen 83 positive common solid cancers

**DOI:** 10.1136/jitc-2022-004713

**Published:** 2022-07-07

**Authors:** Qingyang Li, Wei Hu, Baoyi Liao, Chanchan Song, Liangping Li

**Affiliations:** 1 Department of Clinical Oncology, the First Affiliated Hospital of Jinan University, Guangzhou, China; 2 T Cell Immune Technology Co., Ltd, Guangzhou, China

**Keywords:** antigens, neoplasm, immunotherapy, adoptive, receptors, antigen, T-lymphocytes, epitope mapping

## Abstract

**Background:**

T-cell receptor-engineered T cells (TCR-Ts) have achieved encouraging success in anticancer clinical trials. The antigenic targets, however, were primarily focused on human leukocyte antigen (HLA) A*02:01 restricted epitopes from a few cancer/testis antigens (CTAs) which are not widely expressed in common solid cancers; the tested T-cell receptors (TCRs) were frequently from tumor-infiltrating lymphocytes of old patients and were not assured to have higher avidity. Here, we propose the isolation of high-avidity TCRs against CTAs that are frequently expressed in common solid cancers.

**Methods:**

We selected the CT83 protein, which is frequently expressed in common solid cancers, as a model antigen for screening of its specific TCR. The predicted CT83 epitopes with strong or weak binding to HLA-I molecules, popular in the Chinese population, were integrated into three synthetic long peptides. CT83 reactive CD8+ T cells were stimulated with peptide-loaded dendritic cells (DCs) and sorted using the CD137 biomarker for single-cell sequencing to obtain the paired TCRαβ sequence. The higher frequency TCRs were reconstructed for characterization of the CT83 epitope and for assessment of in vitro and in vivo antitumor activities.

**Results:**

CT83 reactive T cells from young healthy donors (YHDs) were generated by repeated stimulation with DCs and peptides. The single-cell TCR sequencing results of reactive T cells indicated that a single TCR clonotype dominated the paired TCRs. T cells engineered with this dominant TCR led to HLA-A*11:01-restricted recognition of the CT83_14-22_ epitope, with higher avidity. Functional assays showed powerful cytotoxicity in vitro against the targets of several CT83-positive solid cancer cell lines. Furthermore, TCR-Ts showed therapeutic efficacy in three xenograft solid tumor models. The meta-analysis of gene expression of 92 CTAs indicated that most CTAs did not or at low levels in the thymus, which suggested that those CTAs may experience incomplete thymic central tolerance.

**Conclusions:**

High-avidity TCR against CT83 could be isolated from YHDs and efficiently mediate regression of well-established xenograft common solid tumors. The high-avidity TCR repertoire in the peripheral blood of some donors for CT83 and other CTAs provides the basis for the efficient isolation of high-avidity TCRs to target numerous solid cancers.

**Key messages**

- Highly functional T-cell receptors (TCRs) against tumor antigens in common solid cancers are still lacked, meanwhile, most of the target antigens mainly focuses on the human leukocyte antigen (HLA) A*02:01 restricted epitope, which is common in westerners, but few studies have been done on Asians. This study successfully isolated a high-avidity TCR against the CT83 with high expression in solid epithelial cancers and HLA-A11 epitope which is the most common HLA alleles in the Chinese population. Moreover, our approach to isolate the high-avidity TCR from healthy individuals is simple and can be improved to target more CTAs efficiently.

## Background

Adoptive transfer of anticancer T-cell receptor-engineered T cells (TCR-Ts) has become one of the most promising approaches for cancer immunotherapy^1 2^ and has led to complete tumor regression in some patients with melanoma, synovial cell sarcoma and myeloma.^1 3 4^ Currently, clinical trials of TCR-T therapy to target various cancers are rapidly increasing.^5^ Most target antigens in these clinical trials are tumor-associated antigens (TAAs), particularly cancer/testis antigens (CTAs). Many members of CTAs are expressed by a wide range of cancers but are only minimally expressed in normal tissues except germline tissues; hence, they have higher tumor specificity. For instance, the well-studied NY-ESO-1 is a tumor-specific antigen with a higher expression frequency in liposarcoma, neuroblastoma, synovial sarcoma, melanoma, and ovarian cancer,^6^ and is widely used in clinical trials. However, CTA is expressed at a low frequency in many common solid epithelial cancers.^6^

To identify appropriate target antigens for epithelial cancers, we focused our attention on cancer/testis antigen 83 (CT83). This antigen (originally called KK-LC-1) was first discovered by Fukuyama *et al*^7^ as a new CTA in human lung adenocarcinoma and was found to be expressed in many other common epithelial cancers.^8 9^ CT83 is located on chromosome Xq22 and is only expressed in the testes of normal tissues. CT83 is recognized by autologous cytotoxic T cells.^7^ Recently, Stevanović *et al* reported that T-cell receptors (TCRs) recognized that human leukocyte antigen (HLA)-A*01:01 presented epitope CT83_52-60_ from human papillomavirus (HPV)+ cervical cancer patient-mediated complete cancer regression for 46 months.^8^ The National Institutes of Health of the USA conducted phase I clinical trials for CT83-specific TCR.

TCR affinity is a key factor in the development of TCR-T therapies. Successful TCR-T therapy leading to cancer regression mainly depends on T cells with high-affinity TCRs in the optimal functional scope rather than being too low or too high.^10 11^ The biochemical affinity of TCRs is highly correlated with T-cell function (TCR avidity), which can be easily measured by T-cell cytokine assays. It is widely accepted that TCR avidity for self-proteins, including TAAs, is lower than that for non-self-antigens due to central tolerance in the thymus. Although molecular technologies such as in vitro phage display can be used to enhance the affinity of TCR molecules, affinity-enhanced TCRs bear the risk of losing specificity, so that the TCR cross-reacts with other epitopes of human self-antigens.^12^ As human tumor antigens may not be expressed in mice in many cases, the non-tolerant T-cell repertoire might exist in mice for epitopes different from those in humans. Li *et al* established a humanized TCR-HLA mouse (ABabDII) and obtained a HLA-A2-restricted high-avidity TCR (T1367).^13^ However, this ABabDII transgenic strain only expresses one HLA-I allele (A2) and is not suitable for exploring other HLA-restricted TCRs.

Tumor infiltrating lymphocytes (TIL) are a commonly used cell resource for the characterization of anticancer TCRs. The first anti-TAA TCR was isolated from melanoma TILs.^14^ However, they may not be an optimal resource for hunting high-avidity TCRs because patients with cancer are usually elderly and may lose high-avidity TCRs for tumor antigens in the periphery due to immunosenescence.^15–17^ As young healthy individuals normally have a much more diverse TCR repertoire than older individuals, we propose isolating high-avidity TCRs from young peripheral blood mononuclear cells (PBMCs).

Highly polymorphic HLA alleles have distinct regional distribution characteristics. HLA-A*02:01 is one of the most common alleles in Caucasians, whereas HLA-A*11:01, HLA-B*46:01, and HLA-C*01:02 are the most common alleles for each HLA-I locus in the Chinese population. We wondered whether the common HLA-I molecules in Asian people present epitopes of promising CT83.

To explore the discovery of high-avidity TCRs against CTAs, we selected CT83 as a model antigen to establish a technological system for high-avidity TCRs restricted by these three common alleles. Here, we report that a high-avidity TCR restricted by HLA-A*11:01 against the CT83 epitope (CT83_14-22_) was successfully isolated via reverse immunological approaches and single-cell T-cell receptor sequencing (scTCR-seq). This strategy has the potential to become an efficient technique for the rapid characterization of functional TCRs against any CTAs with custom HLA alleles for individualized cancer immunotherapy.

## Materials and methods

### Epitope prediction and peptide synthesis

Epitopes of CT83 for HLA-A*11:01, -B*46:01, and -C*01:02 were predicted using NetMHC V.4.0 and NetMHCpan V.4.0, with a rank threshold for strong binding (SB) of ≤0.5, weak binding (WB) of ≤2.0^18^ and for 8–11 mer peptide length. Three 19-mer peptides (long peptides (LPs)) containing the predicted SB epitopes in the middle were designed for chemical synthesis (Sangon Biotech, Shanghai, China). A group of 9 and 10 mer peptides that shift one amino acid (AA) sequence to the right and overlap eight or nine AAs, except for the last AA, was synthesized to characterize the minimal epitope.

### Generation of CT83 LP-reactive CD8+ T cells

Blood samples were collected from three young healthy donors (YHDs), who were all men in their mid-20s from China, and their HLA genes were genotyped and listed in online supplemental table 2; in their HLA alleles, YHD1, YHD2, and YHD3 contained HLA-A* 11:01, HLA-B* 46:01, and HLA-C* 01:02, respectively.

10.1136/jitc-2022-004713.supp1 Supplementary data

PBMCs were isolated from the peripheral blood using Ficoll-Paque for the separation of dendritic cells (DCs) and T cells using the plastic adherence method. Briefly, the isolated PBMCs were first cultured in tissue culture-treated six-well plates for 2 hours to gain adherent and suspended cells. Adherent cells were used to differentiate into mature DCs by culturing with granulocyte-macrophage colony-stimulating factor (GM-CSF) (1000 IU/mL) and interleukin (IL)-4 (500 IU/mL) for 6 days, followed by a cocktail containing tumor necrosis factor α (TNF-α) (200 IU/mL), IL-1β (1000 IU/mL), and IL-6 (1000 IU/mL) for another 2 days. Then, the final concentration of LPs (5 µg/mL) was added to the mature DCs and incubated overnight. Subsequently, the suspended T cells (1.0×10^7^/well) were cocultured with LP-loaded DCs (1.0×10^6^/well) in six-well plates in T-cell culture medium (RPMI 1640 with 10% human AB serum and 30 IU/mL IL-2) for 8 days as the first round of stimulation.^19^ The LP-loaded DCs were replaced two times every 8 days. After three rounds of peptide stimulation, T cells were cocultured with DC plus LPs overnight. Peptide-reactive T cells were detected using an interferon gamma (IFN-γ) enzyme-linked immunospot (ELISPOT) kit (Dakewe Biotech, Shenzhen, China) and flow cytometry (FCM) analysis with CD3, CD8, and CD137 (4-1BB) antibodies.

### Construction of TCR retroviral vector

The paired TCRα and ΤCRβ genes of the top three TCR clonotypes were linked by the P2A sequence and were murinized in their constant regions. The T2A-linked delta LNGFR (CD271) gene has been included as a universal biomarker for monitoring TCR expression. The expression cassette was cloned into the SalI and EcoRI sites of the retroviral plasmid, pMP71,^13^ to construct pMP71-TCR. The virus supernatant was produced with the packaging lines 293T by transfecting the pMP71-TCR and packaging vectors (pALF10A1 and pcDNA3.1-MLVg/p, a gift from Thomas Blankenstein from MDC-Berlin) using Lipofectamine 3000 (Invitrogen, L3000015).^20^

### TCR transduction of primary T cells

Primary T cells were transduced with the TCR-encoding retroviral supernatant. Briefly, 2 days before viral transduction, 1×10^7^ PBMCs were stimulated in T-cell medium: RPMI 1640 supplemented with 30 ng/mL anti-human CD3 Ab (UCHT1, BioLegend), 30 ng/mL anti-human CD28 Ab (CD28.2, BioLegend), 3000 IU/mL IL-2, and 10% human AB serum. After 48 hours of activation, 1×10^6^ cells/mL lymphocytes were transferred to precoated six-well plates, which were treated with 25 µg/mL recombinant fibronectin fragment (RetroNectin, Takara) for 2 hours, and then the retroviral supernatant was added; the cells were centrifuged at 3000×*g* for 2 hours, and the supernatant was removed.^21^ Twenty-four hours later, the transduced cells were washed with phosphate buffer saline (PBS) and cultured in RPMI 1640 containing 50 IU/mL IL-2% and 10% human AB.

### Cell killing assay

The cytotoxicity of TCR-T was analyzed using the xCELLigence Real-Time Cell Analyser E-Plate 16 system (Agilent, California, USA) according to the manufacturer’s instructions. Target cells (5.0×10^3^) were added to the pre-treated plates, 12 hours later, T cells (effector cells) were added to different effector:target (E:T) ratios ranging from 20:1 to 0.625:1. The cell index, indicating the number of adherent cells, was used to evaluate target cell survival and was recorded every 1 hour.

### Animals and tumor xenograft mouse model

Thirty-six 5-week-old female NCG mice (NOD-*Prkdc^em26Cd52^Il2rg^em26Cd52^*/Gpt) were purchased from GemPharmatech Co. (Nanjing, Jiangsu, China) and maintained under specific-pathogen-free conditions. Mice were randomly divided into three groups to construct different tumor models. Cells (BGC823/A11, MB231/A11, and HCC827; 5.0×10^6^) from three tumor cell lines were subcutaneously injected into the abdomen to establish a cell line-derived xenograft (CDX) tumor implantation model. Seven days later, mice were injected with CT83/TCR1-Ts or control T cells (untransduced T cells (UT-Ts)) followed by 30,000 IU IL-2 once daily for 3 days, and then every 3 days thereafter. During the whole process, tumor size was measured every 3 days and reported as tumor volume (mm^3^), which was calculated using the ellipsoidal formula as follows: =π/6×L×W^2^, where L is the largest tumor diameter and W is the perpendicular tumor diameter. Twenty-seven days later, the mice were euthanized to collect the tumors for subsequent analysis.

### Immunohistochemical (IHC) staining

Tumors from the CDX model were fixed with 4% paraformaldehyde and embedded in paraffin after dehydration. Anti-huCD3 antibody IHC staining was performed to analyze tissue pathology and T-cell infiltration. Sections were imaged using a Nikon DS-U3 microscope at ×200 magnification.

### Statistical analysis

Statistical analyses were performed using GraphPad Prism V.8.0. Statistical significance was calculated using two-way analysis of variance (ANOVA) or one-way ANOVA followed by Tukey’s multiple comparison test, and differences were considered significant at a p value of <0.05.

## Results

### Stimulation of PBMCs by CT83 LP-loaded DCs

**Figure 1:**
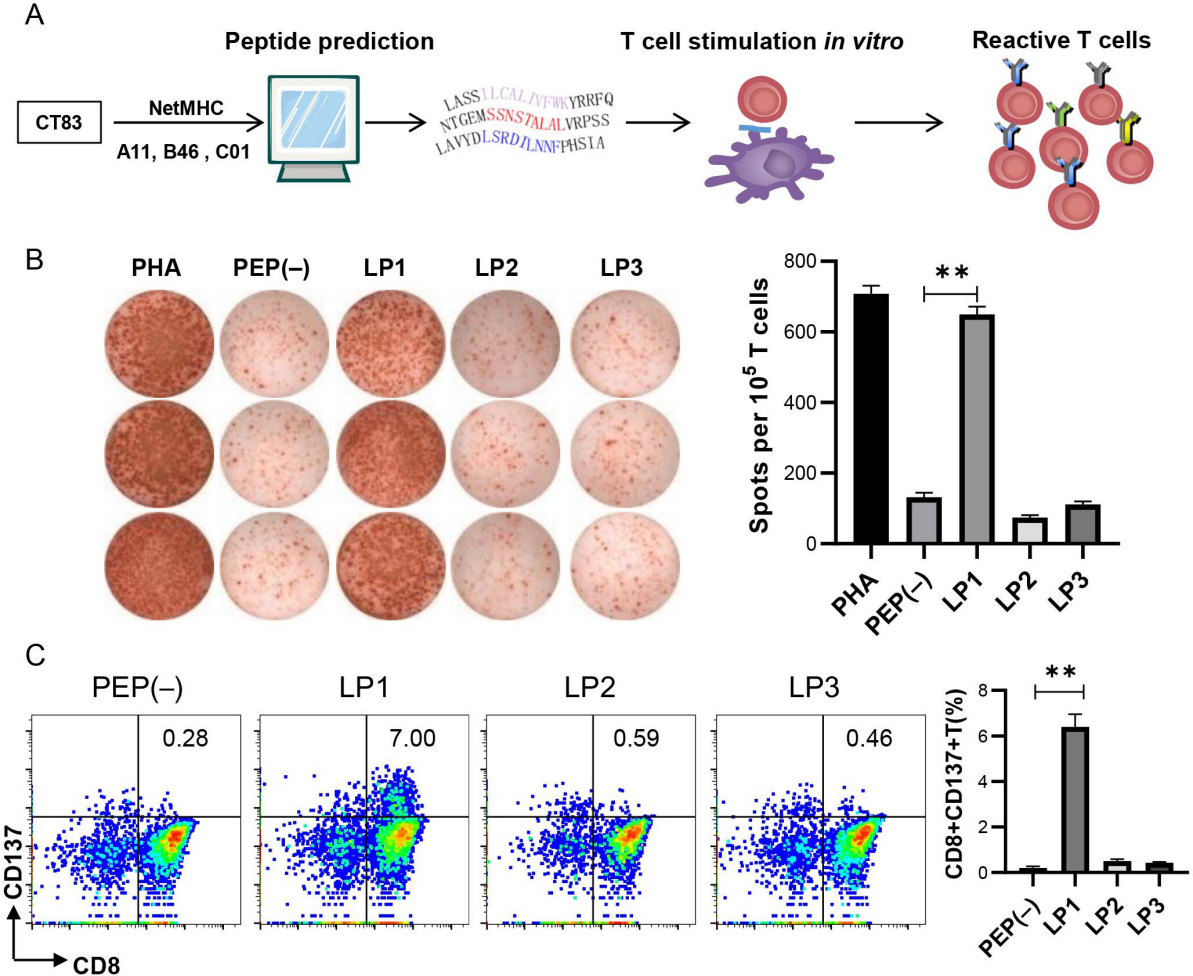
Stimulation of PBMCs by CT83 LP-loaded DCs. (A) Schematic summary outlining the identification of peptide-specific T cells from healthy donors. CT83 epitopes restricted by A11, B46, and C01 alleles were predicted with NetMHC V.4.0 and NetMHCpan V.4.0; three LPs (LP1–LP3) covering the predicted SB epitopes in the middle were synthesized and used to stimulate T cells of PBMCs by LP-loaded autologous DCs for three rounds. (B) Detection of CT83 LP-reactive T cells by IFN-γ. 1×10^5^ stimulated cells were cocultured with CT83 LP-loaded DCs. IFN-γ by secreting T cells were detected IFN-γ ELISPOT assay. Phytohaemagglutinin (PHA) was used as positive control, PBS (PEP−) as negative control. Left panel: scanned image of the ELISPOT wells; right panel: statistical histogram of plots. Columns show mean±SEM of three independent experiments. (C) Detection of CT83 LP-reactive T cells by FCM of CD137. Stimulated T cells were stained with CD3, CD8, and CD137 antibodies and measured by FCM analysis. CD137 upregulation of reactive CD8+ T cells was summarized in the right. Statistical significance was determined with paired two-way analysis of variance followed by Tukey’s multiple comparison test. Data are means±SEM; n=3 independent experiments. **P<0.01. DC, dendritic cell; ELISPOT, enzyme-linked immunospot; FCM, flow cytometry; IFN-γ, interferon gamma; LP, long peptide; PBMC, peripheral blood mononuclear cell; PEP, peptide; PEP−, no peptide; SB, strong binding.

To identify the potential epitopes of CT83 presented by the common HLA-I alleles in Chinese people, we predicted the peptides of CT83 restricted by HLA-A*11:01, HLA-B*46:01, and HLA-C*01:02 alleles and designed an LP-stimulating experimental procedure (figure 1A). The 35 potential epitopes were predicted to have SB or WB to HLA molecules (online supplemental table 1). Based on these three HLA alleles, we designed three 19-mer LPs to cover a total of 20 predicted epitopes. The concrete CT83 LP sequences were LP1, CT83_7-25_ (LASSILCALIVFWKYRRFQ); LP2, CT83_57-75_ (LAVYDLSRDILNNFPHSIA); and LP3, CT83_27-45_ (NTGEMSSNSTALALVRPSS), in which the predicted HLA-A11, B46, and C01-SB epitopes were in the middle of the LPs.

T cells from three YHDs positive for one of the aforementioned three HLA alleles (online supplemental table 2) were in vitro stimulated with the three LP-loaded autologous DCs for three rounds.^22^ After stimulation, T cells from YHD1 showed a markedly IFN-γ response to LP1 (~650 spots/1×10^5^ T cells per well) detected by the IFN-γ ELISPOT assay, but not to LP2 and LP3 (~80 and 100 spots/1×10^5^ T cells per well), which were close to the background(figure 1B). In the FCM analysis of LP1-stimulated T cells from YHD1 cells, approximately 7% of CD8+ T cells upregulated CD137 expression, which was statistically significant (p<0.01) (figure 1C). T cells of YHD2 and YHD3 showed rare or no response to LP1-3 in the IFN-γ ELISPOT assay (online supplemental figure 2) and CD137 upregulation FCM analysis (online supplemental figure 3). In conclusion, these results demonstrated the existence of CT83 LP1-specific T cells in the PBMCs of YHD1.

### Single-cell sequencing of the sorted CT83 LP1-reactive T cells

**Figure 2:**
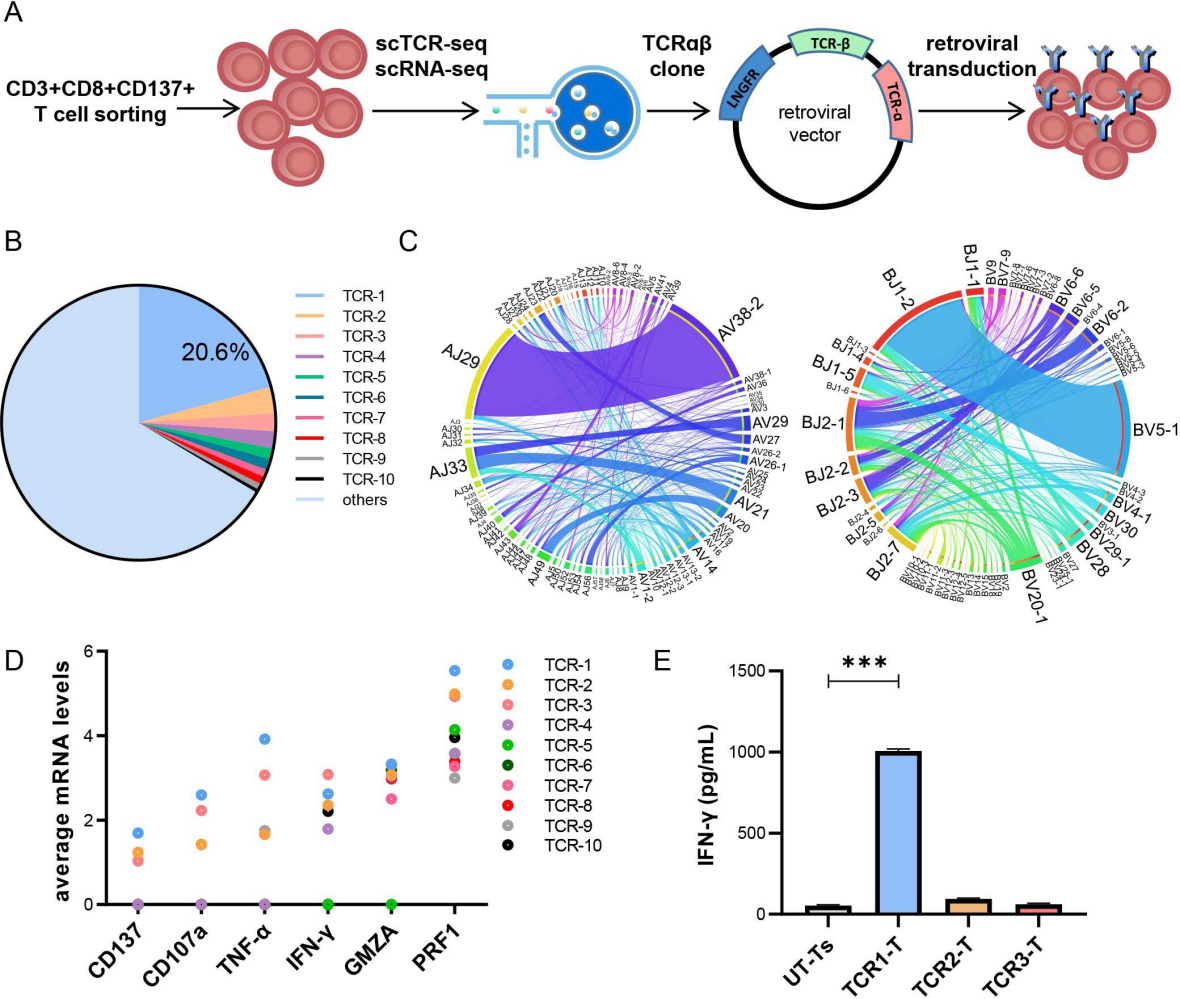
Single-cell sequencing and TCR characterization of the sorted CT83 LP-reactive T cells. (A) Flowchart of CD83 LP-reactive T-cell sorting and single-cell sequencing as well as TCR-T construction. CD3+CD8+CD137+ T cells were sorted for scRNA-seq and scTCR-seq on 10× Genomics Platform. (B) Frequencies of the top 10 TCR clonotypes in scTCR-seq repertoire. TCRs sharing the same protein sequences of both TRA and TRB were defined as a clonotype. (C) The differential TRAV-AJ (left) and TRBV-BJ (right) pairing in scTCR-seq repertoire. Data shown as Circos plots. (D) The mRNA expression levels of CD137, CD107a, TNF-α, IFN-γ, GZMB, and PRF1 in single cells of top 10 TCR clonotypes. (E) LP1 specificity verification of TCR clonotypes. TCR-Ts engineered with the top 3 TCR clones (TCR1–TCR3) were stimulated with LP1-loaded autologous DCs overnight, and the supernatant was analyzed by IFN-γ ELISA. Statistical significance was determined with paired two-wayanalysis of variance followed by Tukey’s multiple comparison test. Data are means±SEM. ***P<0.001. GZMB, granzyme B; IFN-γ interferon gamma; LP, long peptide; PRF1, perforin 1; scTCR-seq, single-cell T-cell receptor sequencing; TCR, T-cell receptor; TCR-T, T-cell receptor-engineered T cell; UT-T, untransduced T cell.

To clone antigen-specific TCRs, we sorted LP1-reactive CD3+CD8+CD137+ T cells for single-cell RNA-sequence (scRNA-seq), scTCR-seq, and TCR-T construction (figure 2A). Analysis of the sequencing data showed that 6456 cells contained paired TCR α and β chains. From the TCR repertoire analysis of these paired TCRαβ sequences, we found the top 10 TCR clonotypes (figure 2B and online supplemental table 3). TCRαβ V-J usage in the scTCR repertoire is shown in figure 2C (TRAV-AJ in figure 2C, left panel, and TRBV-BJ in figure 2C, right panel), and the dominant TCR clonotype is TRAV38-2/AJ29 pairing with TRBV5-1/BJ1-2 (20.6% of all paired TCRαβ clones). The expression levels of immune effector factors, including CD137, CD107a, TNF-α, IFN-γ, granzyme B, and perforin-1, were analyzed in the scRNA-seq data. A highly positive correlation was observed between TCR clone frequency and the expression levels of these factors (figure 2D). We chose the top three clones (TCR1–TCR3) to reconstruct the complete TCRαβ chains for the preparation of the retroviral expression vector CT83/TCR1-3 and to characterize the antigen specificity of TCRs. A large amount of IFN-γ was detected by ELISA in the supernatant of CT83/TCR1-Ts but not in those of CT83/TCR2-Ts and CT83/TCR3-Ts against LP1-loaded autologous mature DCs (figure 2E). Thus, CT83/TCR1-T cells recognized an epitope from CT83 LP1 and were used in subsequent experiments.

### Identification of CT83 LP1 epitope and its HLA restriction

**Figure 3:**
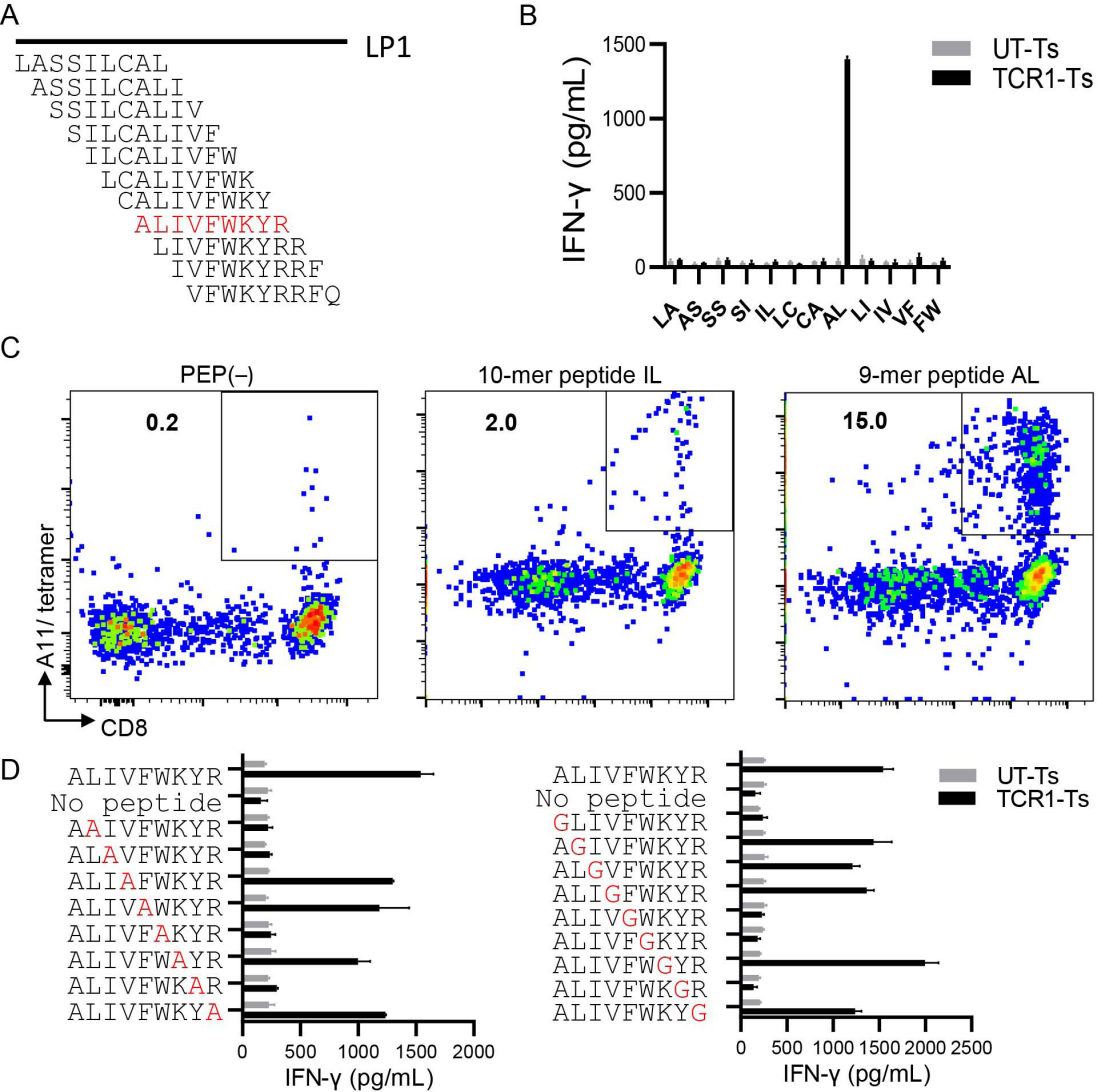
Epitope characterization of CT83 LP1 for CD83/TCR1 recognition. (A) A panel of overlapping 9-mer peptides for the full sequence of LP1. The 9-mer peptides were named with the first two letters and were used to load T2/A11 cells. The AL (red color) peptide was the proved epitope in LP1. (B) IFN-γ releasing of CT83/TCR1-Ts stimulated with peptide-loaded T2/A11 cells and detected via ELISA assay. (C) Representative FCM data for A11 tetramer staining. Right panel: 9-mer AL peptide exchanged A11 tetramer; middle panel: 10-mer IL peptide exchanged A11 tetramer; and left panel: PEP− exchanged A11 tetramer. (D) Residue scanning of the AL epitope by substitution with alanine (left panel) and glycine (right panel). The substituted peptides were evaluated for stimulation to TCR1-T cells for IFN-γ secretion detected by IFN-γ ELISA. The substituted alanine (A) and glycine (N) were labeled with red color. CT83/TCR1-T, CT83/TCR1 engineered T cell; FCM, flow cytometry; IFN-γ, interferon gamma; LP, long peptide; PEP, peptide; PEP−, no peptide; UT-T, untransduced T cell.

CT83 LP1 contained five predicted high-affinity epitopes (<400 nM) restricted by the HLA-A*11:01 allele. To characterize the A11 restricted epitope, we stimulated CT83/TCR1-Ts with T2/A11 cells (online supplemental figure 6A), separately loaded with a panel of 10 overlapping 10 mer peptides covering CT83 LP1. No obvious IFN-γ secretion was observed in this experiment (data not shown). Subsequently, we tested a panel of 11 overlapping 9 mer peptides (figure 3A) and observed a strong T-cell response to the 9 mer peptide AL (ALIVFWKYR) (figure 3B). To confirm the CT83/TCR1 specificity, we constructed a CT83/HLA-A*11:01 tetramer with a 10 mer ILCALIVFWK (CT83_11-19_) peptide and a 9 mer ALIVFWKYR (CT83_14-22_) from an exchanged tetramer of HLA-A*11:01 (figure 3C, middle and right panels). FCM analysis showed that both tetramers stained CT83/TCR1-T cells, but the staining of CT83_14-22_/HLA-A*11:01 tetramers (15%) (figure 3C, right panel) was much higher than that of the CT83_11-19_/HLA-A*11:01 tetramers (2.0%) (figure 3C, middle panel). Therefore, we conclude that CT83/TCR1 preferentially binds to the 9 mer AL epitope presented by HLA-A*11:01.

Next, we identified the AA motif within the AL peptide responsible for T-cell recognition by changing each AA position to alanine (figure 3D, left panel) or glycine (figure 3D, right panel). When compared with the wild-type AL epitope, alanine substitutions at positions 2, 3, 6, and 8 and glycine substitutions at positions 1, 5, 6, and 8 resulted in an 80% reduction in IFN- release. Alanine at position 1 of the AL peptide could only be checked in the glycine substitution assay. Based on the substitution results for AA residues A and G, we selected the common replacement residues 6 and 8 that have an effect on the reaction. Because sequence position 1 is A, it can only be verified in the glycine substitution scheme, so our judgment of this position comes from the substitution result of G. 1, 6, and 8 were inferred to be the most essential non-anchor residues for TCR recognition. Therefore, AAs at positions 1, 6, and 8 (AxxxxWxYx motif) were required for specific antigen recognition by CT83/TCR1-transduced T cells. Based on this motif, we evaluated the possible off-target effects of CT83/TCR1 by analyzing its cross-reactivity with potential epitopes from other human proteins. The results were then analyzed using the online tool Expitope V.2.0 to screen for motif similarities in the human proteome. We found that only one human epitope, with up to four mismatched AAs, exists within the human proteome. CT83/TCR1-Ts was tested for recognition of the candidate peptides in a coculture assay; recognition was not detected (online supplemental figure 7). Thus, CT83/TCR1 did not demonstrate detectable cross-reactivity against human peptides in vitro,^13^ indicating that CT83/TCR1 is highly specific for the CT83_14-22_ epitope without any mimic epitope in the human proteome, as well as an extremely low off-target effect and a good safety profile for its TCR-Ts.

### Functional characterization of CT83/TCR1

**Figure 4:**
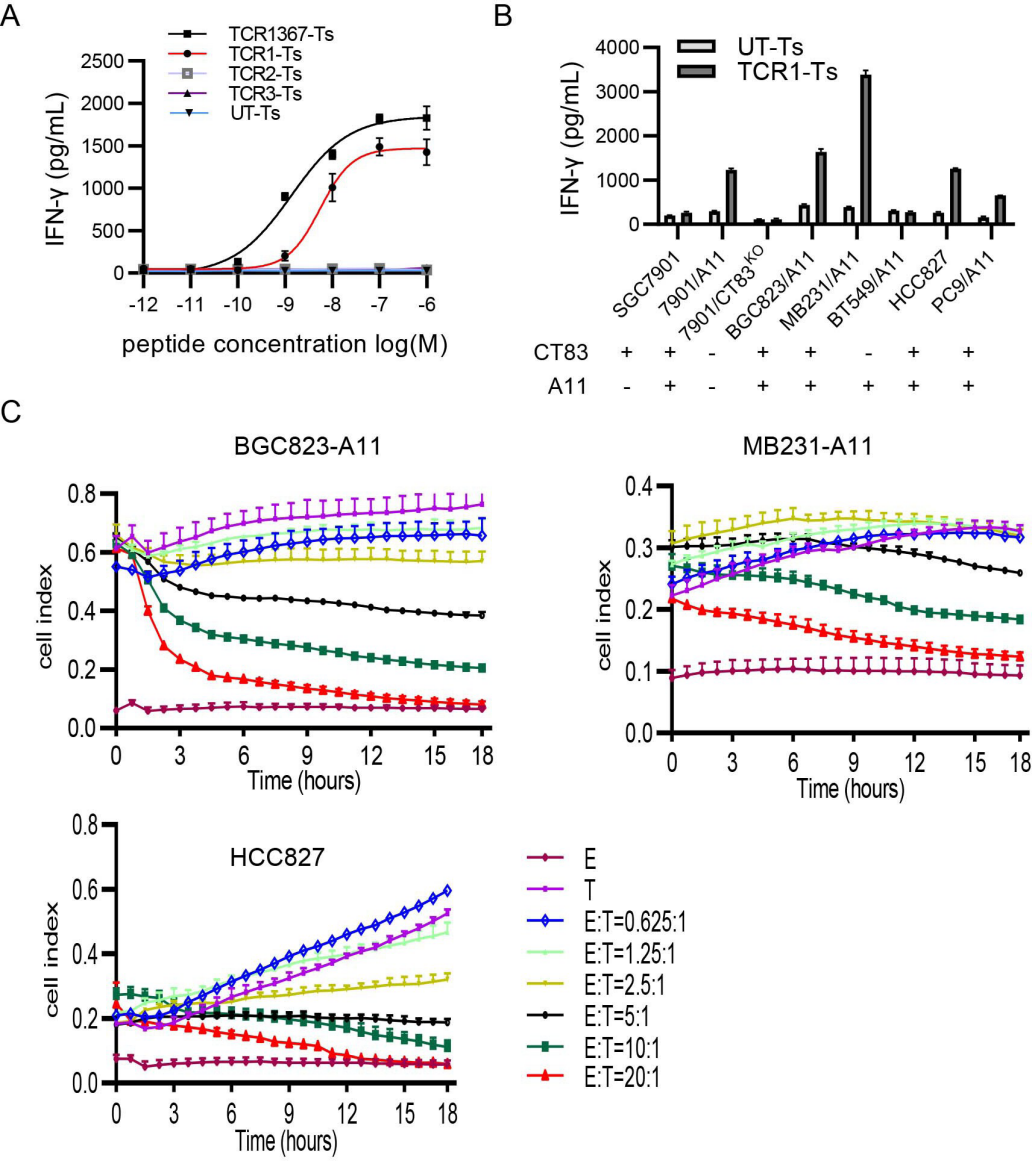
Characterization of CT83/TCR1 functional avidity. (A) Functional measurement of TCR1: IFN-γ production of CT83/TCR1-Ts stimulated with log-diluted AL-loaded T2/A11 was tested with IFN-γ ELISA. TCR2 and TCR3 were as negative control, while TCR1367 (recognizing MAGE-A1 with HLA-A*02:01 restriction) was selected as reference. Data are means±SD (n=3 replicates). (B) IFN-γ production of CT83/TCR1-Ts on stimulation with human tumor cells for 18 hours. Tumor cell lines from 3 kinds of solid tumors: STAD (SGC7901, BGC823), LUAD (HCC827, PC9), and BRCA (MB231, BT548) whose expression of CT83 and HLA-A*11:01 was shown on the down (−, negative; +, positive). Data are means±SD (n=3 replicates). (C) CT83/TCR1-Ts mediated killing of three tumor lines. BGC823/A11, MB231/A11 and HCC827 cells (target cells (T) were incubated in a 16-well chamber overnight. Various amounts of transduced or non-transduced T cells (effector) were added into wells to form E:T ratios ranging from 0.625:1.0 to 20.0:1.0. Cell index was measured with the xCELLigence Real-Time Cell Analyzer. Data are means±SD (n=3 replicates). CT83/TCR1-T, CT83/TCR1 engineered T cell; E:T, effector:target; IFN-γ, interferon gamma; TCR, T-cell receptor; UT-Ts, untransduced T cells.

To assess the functional avidity of CT83/TCR1, we performed experiments for T2/A11 stimulation loaded with a titrated concentration of cognate peptides, the recognition of several solid tumor cell lines and the cytotoxicity of three representative cancer cell lines with CT83/TCR1-T cells. IFN-γ production by the CT83/TCR1-Ts in the reaction to T2/A11 cells loaded with titrated concentrations of AL peptide showed that CT83/TCR1-T cells recognized the AL peptide at concentrations as low as 10^−9^ M (EC50:5.5×10^−9^ M) (figure 4A), which was very close to the high-avidity T1367 used as a positive control in parallel experiments.^13^

To study whether CT83/TCR1-Ts could recognize the naturally cognate antigen from cancer cell lines, we first checked CT83 and A11 expression in eight cancer cell lines (online supplemental figure 8) and measured the T-cell response to these cell lines (figure 4B). CT83/A11 double-positive cell lines (HCC827, SGC7901/A11, BGC823/A11, MB231/A11, and PC9/A11) stimulated CT83/TCR1-Ts to secrete various amounts of IFN-γ, but single CT83-positive (SGC7901), single A11-positive (BT549), and double-negative (SGC7901/CT83^KO^) cell lines could not be recognized by CT83/TCR1-Ts. This evidence indicates that CT83/TCR1 specifically recognizes cancer cell lines from three common solid cancers: stomach, breast, and lung.

To observe dynamic cell killing, we performed a real-time cytotoxicity assay for CT83/TCR1-Ts against three representative CT83/A11-positive cell lines from stomach, lung, and breast cancers. When the E:T ratio reached 10:1 or higher, most tumor cell lines (BGC823/A11, MB231/A11, and HCC827/A11) were killed by CT83/TCR1-Ts during coculture overnight (figure 4C), among which BGC823/A11 was one of the most sensitive lines. These data indicated CT83/TCR1 TS had target specificity and a killing effect against naturally processed antigens.

### In vivo antitumor effect of CT83/TCR1-Ts

**Figure 5:**
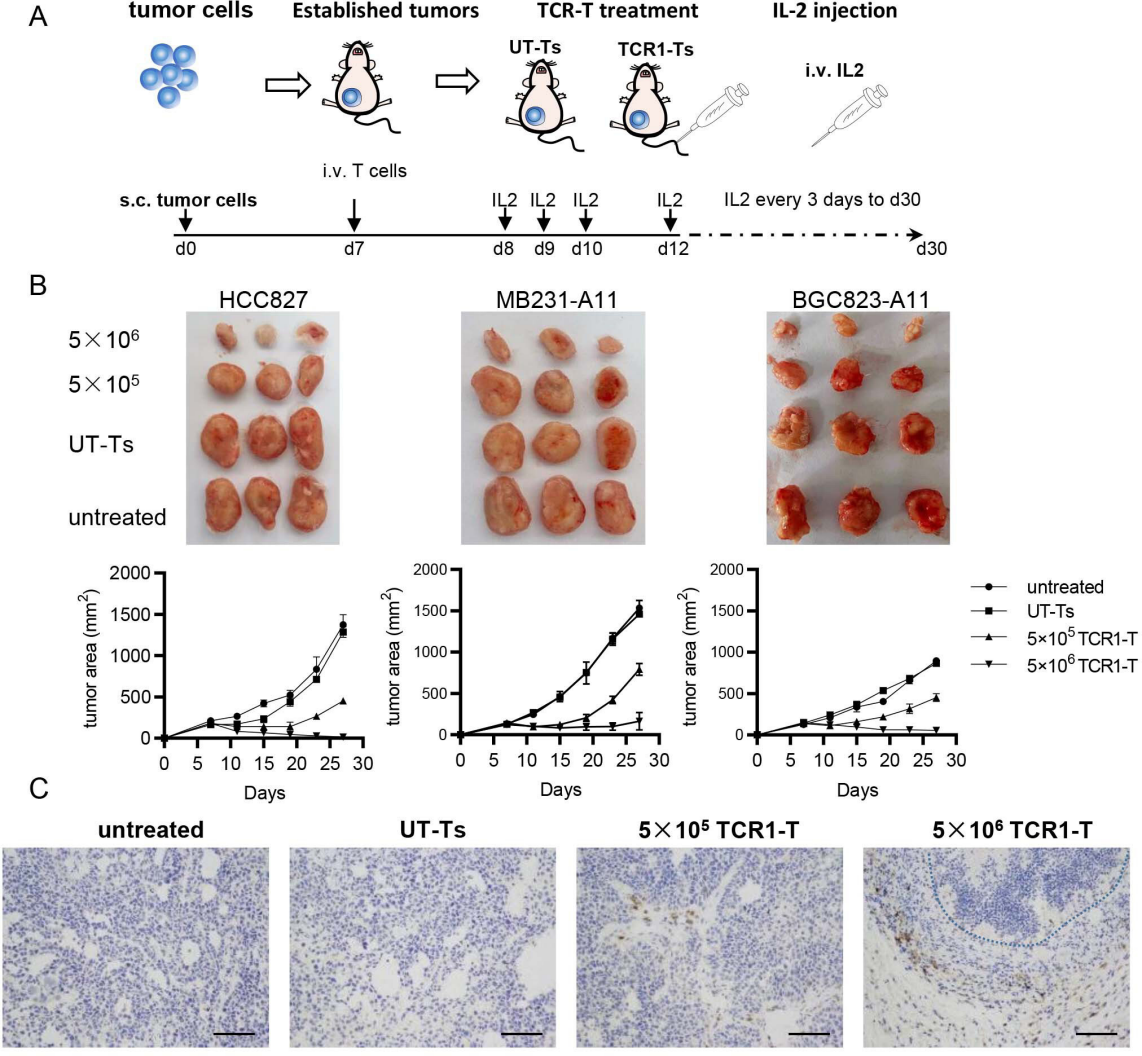
Antitumor activity of CT83/TCR1-Ts in CDX mouse model. (A) Flowchart of CT83/TCR1-T cell therapy in CDX NCG mice model. The mice with the 1 week established HCC827, MB231/A11, and BGC823/A11 tumor CDX were treated with CT83/TCR1-Ts followed with 30 000 IU IL-2 for 27 days. (B) Treatment of the three CDX tumor models. The three photos on the upper panel show the tumors extracted at day 27. Each group contained three mice. The tumor growth curve displayed in the down panel. Data are means±SEM (n=3 mice/group). (C) Human T-cell infiltration in tumor tissue. T cells in tumor tissue sections from HCC827 were detected by IHC staining of T-cell marker CD3 with anti-huCD3 antibody. Untreated (without injection with T cells) and UT-Ts (injected with UT-Ts) were used as negative controls. The experimental treatment groups were injected with different doses of T cells (5×10^5^ and 5×10^6^ TCR1-Ts, respectively). Dotted line: necrosis area. Brown staining: CD3+ T-cell area. Scale bars, 100 µm. CT83/TCR1-T, CT83/TCR1 engineered T cell; IHC, immunohistochemical; IL, interleukin; TCR, T-cell receptor; UT-T, untransduced T cell.

To investigate the potential of CT83/TCR1-Ts to mediate the regression of cancers in vivo, we employed immunodeficient NCG mice to establish murine CDX models. One week later, well-established CDX tumors were treated with CT83/TCR1-Ts and IL-2 adjuvant via intravenous injection (figure 5A). Compared with untreated and UT-T controls, all mice treated with 5×10^5^ CT83/TCR1-Ts displayed tumor regression, and the tumors regressed more obviously at a higher dose of 5×10^6^ CT83/TCR1-Ts, showing a tumor growth curve and tumor samples extracted on day 27 (figure 5B).

Finally, IHC staining of the treated tumors (from representative cancer cell line HCC827) revealed extensive CD3+ T-cell infiltration in the CT83/TCR1-T groups (figure 5C, the two photos on the right), while almost no T-cell infiltration was observed in the untreated and UT-Ts groups (figure 5C, the two photos on the left). In the tumor regression group with 5×10^6^ CT83/TCR1-Ts, dark-stained necrosis of tumor cells was clearly observed in the middle of the T-cell infiltration (figure 5C, photo on the right). IHC staining of tumors from MB231/A11 and BGC823/A11 is shown in online supplemental figure 9. These findings support that CT83/TCR1-T therapy could result in tumor regression of human stomach, lung, and breast cancers in CDX models.

### CTA bioinformatics analysis for CTA expression on cancer and thymus

**Figure 6:**
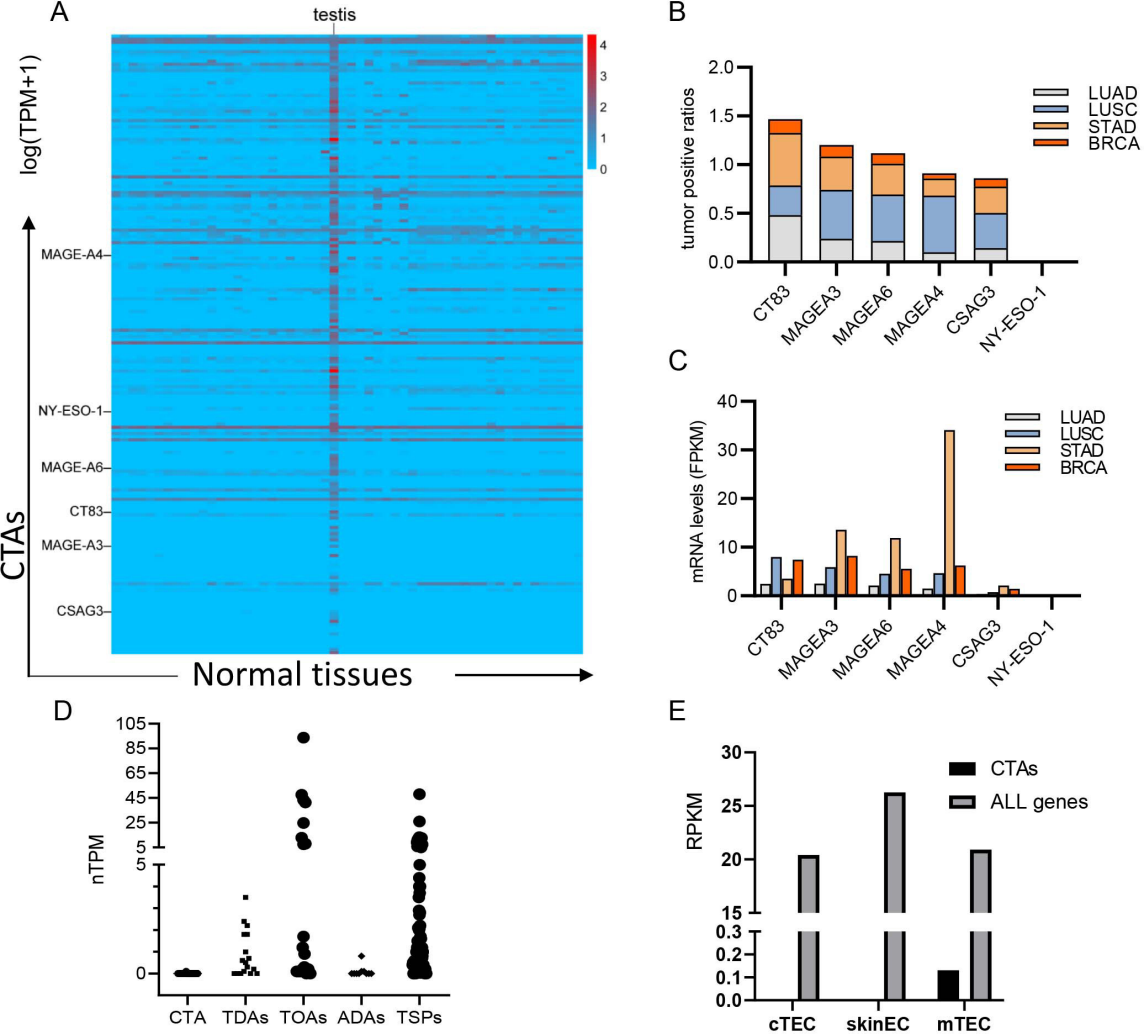
Bioinformatics analysis of the CTAs. (A) Heat map of the mRNA expression of CTAs in normal organs/tissues. Raw data of 198 CTAs were collected from GTEx. A total of 92 candidate CTAs was screened based on specific expression in the testis. (B) The positive expression ratios of the top 5 CTAs in LUAD, LUSC, STAD, and BRC. (C) The FPKM value of mRNA relative expression levels of these five CTAs in common tumors. NY-ESO-1 was used as a reference in (B, C). (D) Thymic expression data of 92 CTAs, 17 TDAs, and 19 TOAs as well as 11 ADAs and 77 TSPs. (E) Meta-analysis RNA-seq data of gene expression of the sorted murine thymic epithelial cells. The RPKM values are the average RPKM of CTAs and the original thymic transcriptome (ALL genes, seen in Shida *et al*^27^) on each group of cTEC, skinEC, and mTEC. The mouse CTA list is seen in online supplemental table 4. ADA, autoimmune disease antigen; CTA, cancer/testis antigen; cTEC, cortical thymic epithelial cell; GTEx, Genotype-Tissue Expression; mTEC, medullary thymic epithelial cell; TDA, tumor differentiation antigen; TOA, tumor overexpressed antigen; TSP, tissue-specific protein.

To extend the application of our experiments, we systematically analyzed the gene expression profiles of 224 functional CTAs (online supplemental table 4, file 224 CTAs) and created a heat map of gene expression for 198 CTAs using available data from the Genotype-Tissue Expression (GTEx) database (GTEx Portal) (figure 6A and online supplemental table 4, file 198 CTAs). When screened under strict conditions, 92 candidate CTAs specifically expressed in the testes, but 51 samples did not in normal tissues and cells. Subsequently, we used The Cancer Genome Atlas data of 24 types of cancers to validate the expression of 92 candidate CTAs in these common solid tumors. Statistics data of positive expression ratios revealed that five CTAs (CT83, MAGEA3, MAGEA6, MAGEA4, and CSAG3) were frequent in these common cancers (figure 6B, C); among them, CT83 had the highest positive expression ratios overlying in these four common solid cancers: 54% stomach adenocarcinoma, 48.3% lung adenocarcinoma, 30% lung squamous cell carcinoma, and 14% breast invasive carcinoma, as well as in the four common solid cancers: 45% esophageal carcinoma, 21% pancreatic adenocarcinoma, 19% cervical squamous cell carcinoma and endocervical adenocarcinoma, and 15% bladder cancer (online supplemental table 4, file top 5 CTA-positive ratios). As for relative expression levels in these common tumors (figure 6B, C, and online supplemental table 4, file top 5 CTA expression level), CT83 was in the middle ranking among the five CTAs but much higher than that of NY-ESO-1. Based on these analyses, we conclude that CT83 is one of the most appropriate target antigens for adoptive TCR-T therapy.

10.1136/jitc-2022-004713.supp2 Supplementary data

To explore the possible mechanism of the existence of high-avidity TCRs for CTAs in the peripheral blood of healthy donors, we analyzed the thymic expression data of various tumor antigens, including CTAs, tumor differentiation antigens (TDAs), and tumor overexpressed antigens (TOAs) from the human platelet antigen (HPA) database.^23^ As a reference control, we also included a group of tissue-specific proteins (TSPs) and autoimmune disease antigens (ADAs) listed in Gotter J *et al*^24^ and Lv H *et al* studies.^25^ The evidence that most TSPs could be detected in the HPA dataset demonstrated that the HPA data for the thymic expression of various proteins was reliable. We found that 7/11 (63.6%) autoimmune antigens could not be detected, and only 5/77 (6.5%) tissue-specific genes could not be detected in the thymus from the HPA database (online supplemental table 4). The average expression value for autoimmune self-antigens was very low, 0.14 normalized transcripts per million (nTPM); however, it was 5.58 nTPM for tissue-specific genes (figure 6D).

As for CTAs, we discovered that CT83 was not detected in adult and infant thymuses. Furthermore, we searched the thymic expression data of 92 CTAs and discovered that most CTAs were not expressed in the thymus, and only a few CTAs (7/92) were expressed slightly (0.1 nTPM). In contrast, the thymus expresses a variety of TDAs as well as TOAs.

To analyze the gene expression of mouse CTAs (MAGE and PRAME gene families as well as a few others) on cortical thymic epithelial cells (cTECs) and medullary thymic epithelial cells (mTECs), we extracted the RNA-seq data of the sorted mouse thymic epithelial cells published by St-Pierre *et al*^26^ and found that 30% of CTAs were completely absent on mTECs, 70% expressed at low levels (average 0.132 RPKM) (figure 6E and online supplemental table 4). Taken together, this evidence suggests that most CTAs might be expressed at low levels or absent in the thymus at the time of the experiment.

## Discussion

Successful TCR-T therapy depends on two main factors: suitable tumor-specific target antigens and their high-avidity TCRs. To develop a safe and effective TCR-T therapy for CTAs, the top priority is to select suitable and widely expressed target antigens. We screened 92 candidate CTAs expressed in the testis, but not in normal tissues, from 198 CTAs. Five CTAs were identified to be frequent in the most common solid cancers, and CT83 expression had the highest positive rate among the five CTAs. Previous studies on CT83 expression in cancer showed similar results; CT83 is highly expressed in gastric,^27 28^ triple-negative breast,^29^ and non-small cell lung cancer.^30^ This bioinformatics analysis and experimental studies have demonstrated that CT83 is one of the most suitable targets for solid cancers.

Higher immunogenicity of tumor antigens is essential for successful immunotherapy. Several previous reports demonstrated that the CT83 antigen could stimulate T-cell responses: (1) HLA-B*15:07 restricted cytotoxic T lymphocyte (CTL) clone H1 responded to CT83_76-84_^7^; (2) HLA-A*02:01-restricted CT83_66–74_ epitope was characterized in A2 transgenic mice^31^; and (3) a CD8+ T cell-derived and HLA-A*01:01–restricted TCR could recognize the CT83_52-60_ epitope. This evidence demonstrated that CT83 is immunogenic.

Based on these previous studies, we focused on the discovery of CT83-specific TCRs restricted to the top three HLA-I alleles in the Chinese population from the PBMCs of YHDs and successfully screened out A11 restricted CT83/TCR1, which recognized the CT83_14-22_ epitope. To estimate the extent of functional avidity of CT83/TCR1, we studied high-avidity TCRs claimed in the literature with avidity data from stimulation with a log concentration of peptides and found that the half-maximal effective concentration (EC50) was a comparable factor among different TCRs. The first reported high-avidity TCR (DMF5) for MART-1 was isolated from TILs of a patient with melanoma with an EC50 of approximately 5.0×10^−8^ M.^32 33^ T1367 (EC50 approximately 2.0×10^−9^ M) isolated from TCR-HLA transgenic mice had higher functional avidity than those from patients’ TILs.^13^ Based on the functional avidity of these two high-avidity TCRs, we defined the EC50 of the natural TCRs with high avidity in the range of 10^−8^ to 10^−9^ M.

Based on this standard, we compared CT83-specific TCRs. As reported, the EC50 of the first CT83-specific TCR identified from a 69-year-old patient with lung cancer by Fukuyama *et al* was approximately 8.7×10^−9^ M,^7^ and Stevanović *et al* isolated CT83-specific TCR from TILs of patients with HPV+ metastatic cervical carcinoma; the EC50 of this TCR was about 5×10^−8^ M. Our functional avidity assessment indicated that the EC50 of CT83/TCR1 was approximately 5.0×10^−9^ M. Although these three CT83-specific TCRs were all in a range of high avidity, the avidity of CT83/TCR1 from a young donor was approximately 2-fold to 10-fold higher than that of the patients with cancer. This evidence demonstrated that peripheral T cells of young donors are the optimal resource for the high-avidity TCR repertoire of CTAs.

Why was it easier to isolate high-avidity TCRs from healthy individuals or patients with cancer against CT83 in these three studies? The high affinity of the TCR repertoire in the periphery is highly dependent on thymic negative selection, in which CD4+ and CD8+ thymocytes with high-affinity TCRs for self-antigens, including TAAs, are depleted by clonal deletion in the thymic medulla.^34 35^ For example, Theobald *et al*^36^ demonstrated tolerance of the thymus to TAA p53. The avidity of CTLs obtained from p53 WT mice was 10-fold lower than that obtained from p53 knockout mice. In a mouse model of murine CTA P1A, this protein was found to be expressed in mTECs,^37^ and Huijbers *et al* reported the existence of minimal tolerance to this mouse CTA.^38^

After passing the positive selection at the cTECs, the CD4 and CD8 double-positive or single-positive thymocytes enter the medulla, a dense reticular network, which consists of mTECs, DCs, and B cells. The autoimmune regulator (AIRE) transcriptional regulator of mTECs endows the promiscuous gene expression of TSPs and forms an entire mosaic self-ligandome, which educates CD4 and CD8 SP thymocytes and removes self-reactive thymocytes. If this network is dense and impenetrable, self-reactive T cells for autoimmune disease or CTAs would not appear in the periphery. However, there are some leaky loopholes in this ‘self-peptide’-presenting network. Lv *et al*^25^ showed that the lack of self-antigen myosin heavy chain six in mTECs correlated with a high frequency of cognate autoreactive T cells and autoantibodies in the periphery.

We analyzed the thymic gene expression of common 11 ADAs and 92 CTAs (figure 6D) from the HPA database. Both genes were expressed negatively or at a low level in the thymus, although the possibility of higher expression in mTECs could not be excluded. RNA-seq data from purified mouse mTECs demonstrated that the thymus has a small leaky loophole for the negative selection of some CTAs (figure 6E and online supplemental table 4).

This observation is inconsistent with the previous report. Gotter *et al* 24 have shown that several CT antigens, such as MAGE-A1, MAGE-A3, MAGE-A4, and NY-ESO-1, were expressed on purified mTECs of the thymus, as detected by RT-PCR. However, with close data inspection, we found that the RT-PCRs for CT antigen were low or negative in some samples, and TDAs (Mart-1 and tyrosinase) expressed a slightly higher level and were less variable than CTAs.

The low-level expression of CTAs may lead to incomplete thymic central tolerance in some individuals; therefore, high-avidity T cells would appear in their periphery. We also observed that T cells from some HLA-A11-positive donors responded to CT83 LP1 at low levels (data not shown); thus, these individuals should have stronger central tolerance for CT83. Although the higher-affinity TCRs for some CTAs appear in the periphery, the peripheral tolerance mechanism (eg, the testis cells lack HLA expression) would prevent the attack to self-normal testis tissues by the T cells with higher avidity TCR for those CTAs. However, these T cells may have a potential function in preventing cancer if CTAs are upregulated at the early stage of carcinogenesis.

The isolation of higher avidity TCRs from individuals with less central tolerance and the in vitro enhancement of TCR affinity to achieve the optimal avidity TCR for CTAs will help target the CTA-positive cancers.

Tumor-specific expression of CT83 ensures the safety of T-cell treatment to target this antigen. A clinical study of TIL infusion containing CT83-specific T cells for CT83 proved its safety.^8^ No acute toxicity related to cell infusion or adverse autoimmune events was observed. For the A11 restricted CT83 epitope, ALIVFWKYR, our alanine and glycine substitution assay demonstrated that cross-reactivity to other human proteins did not exist. In summary, CT83 is a safe and immunogenic target antigen for the development of immunotherapy for many solid tumors.^9^

## Conclusions

This study reported the discovery of an epitope of CT83 presented by HLA-A*11:01 and its specific TCR in healthy young people. CT83/TCR1-Ts demonstrated high avidity recognition and killing in various cancer cell lines in vitro, as well as inhibition of tumor growth in animal models in vivo. In view of the high tumor specificity, pan-cancerous characteristics, and high expression level of CT83, it is worth further studying the antitumor effect of CT83/TCR1-Ts in clinical trials of immunotherapy. Moreover, our approach to isolate the high-avidity TCR from healthy individuals is simple and effective and can be improved to target more CTAs efficiently.^39^

## Data Availability

Data are available in a public, open access repository.
