# Supplementary material for: Natural high-avidity T-cell receptor efficiently mediates regression of cancer/testis antigen 83 positive common solid cancers
Source: J Immunother Cancer. 2022 Jul 7;10(7):e004713. doi: 10.1136/jitc-2022-004713 (PMC9263944; doi:10.1136/jitc-2022-004713)
Supplement: Supplementary data [file jitc-2022-004713supp001.pdf]

Supplementary methods

Cell lines and modifications

The 293T and T2 cell lines were obtained from ATCC. 293T and T2 cells were grown in RPMI 1640 medium (Gibco) supplemented with 10% FBS. Six solid cancer cell lines used in the study were grown in DMEM supplemented with 10% FBS: SGC7901 and BGC823 from stomach cancer, MB231 and BT549 from breast cancer, and HCC827 and PC9 from lung cancer. HLA-A*11:01 (A11)-negative cells (SGC7901, BGC823, MB231, BT549, and PC9) were modified with a lentiviral A11 expression vector to obtain A11-positive cells (abbreviated as SCG7901/A11, BGC823/A11, MB231/A11, BT549/A11, PC9/A11; SGC7901 was targeted by CRISPR/Cas9 technology to gain CT83 knockout cells, SGC7901/CT83^{KO}. To establish A11-positive and TAP-negative cells, T2 cells were first knocked out for the B2M gene (online Supplemental Figure 6A) and then transduced with the lentiviral A11 expression vector to generate the T2/A11 cell line.

Flow cytometry analysis.

The following conjugated antibodies were purchased from BioLegend and used for staining at 1:100 dilution: anti-CD3-APC, clone UCHT1; anti-CD3-PE/CY7 clone UCHT1; anti-CD8-PE/CY7, clone HIT8a; anti-CD137-PE/CY5, clone 4B4-1; anti-CD271-PE, clone ME20.4; anti-HLA-A/B/C-PE, clone W6/32. QuickSwitch™ Quant HLA-A*11:01 Tetramer Kit (MBL, Japan) were used to prepare peptide-HLA-A*11:01-PE tetramer. FCM analysis was run in BD FACSCanto II, while cell sorting

was conducted in the BD FACSAria II. The data were processed using FlowJo software and the gating strategy is shown in Supplemental Figure 5.

Single cell TCR sequencing.

$CD3^+CD8^+CD137^+$ T cells (2×10^5) were sorted from above peptide-reactive T cells and subjected to single cell RNA sequencing (scRNA-seq) (10x Genomics, USA). The sequencing data were processed for identification of TCR clonotype by alignment and annotation using the Cell Ranger pipeline (version 2.1.0).

CRISPR/Cas9 gene editing.

To disrupt the endogenous HLA-I expression of TAP-deficient T2 cells (HLA-A2+), we conducted CRISPR/Cas9 B2M gene editing experiment to target "GAGTAGCGCGAGCACAGCTA" DNA sequence of human B2M gene. Briefly, 6.25 μg Cas9 protein (Invitrogen, A36498) and 37.5 pmol sgRNA were mixed to form a B2M ribonucleoprotein complex (B2M-RNP) and added into 100 μL Nucleofector buffer (Lonza). 1×10^6 T2 cells were transfected with the above B2M-RNP/ Nucleofector solution by electroporator 2B Nucleofector with A-030 program. HLA-I expression was detected by staining with anti-HLA-ABC antibodies (Biolegend, 311406). Pure HLA-I negative cells were enriched by sorting with FACS AriaII (BD) and limiting dilution method. To establish the CT83 negative tumor cell line, we constructed a KO plasmid (the structure diagram is shown Supplemental Figure1A containing CT83 sgRNAs and Cas9 coding sequence, the sgRNA target sequences are respectively "TGGCTAAAATATACTTACTG"and"AGGCGGTACTAAGTGCCGCC". 1×10^6

cells of SGC7901 were transfected with the CT83 KO plasmid by electroporator 2B Nucleofector. The inclusion of the EGFP and Puro genes in the KO plasmid allowed to monitor the transfection under a fluorescence microscope and enrich the CT83 negative cells (SGC7901 $CT83^{KO}$) by treating with puromycin.

Western blot analysis.

CT83 protein expression of tumor cell lines was analyzed by standard western blotting with cell lysate prepared by Tissue and Cell Total Protein Extraction Kit (Applygen, Beijing, China). The blotting membrane was detected using anti-huCT83 mouse monoclonal antibody (CL4762, AB219971, Abcam) (1:1000) and goat-anti-mouse IgG conjugated with horseradish peroxidase (HRP) (BS12478, Bioworld) (1:40000); GAPDH was used as normalized control. The protein bands were detected by ECL Plus Western Blotting Substrate (Boster Bio, CA, USA) and recorded on an X-ray film, the density of bands was determined with Image J software.

HLA gene transfer.

To introduce exogenous HLA-I gene into T2-$B2M^{KO}$ cells, we constructed lentiviral HLA-A*11:01 expression vector. cDNAs of human B2M and HLA-A*11:01 were fused together via (Gly4Ser)3 linker to form 1436bp DNA fragment, which was inserted into the NotI and BamHI sites of CSII-EF-MCS-IRES2 vector to construct CSII-EF-hB2M-A11. For lentiviral transduction. Briefly, a total of 1×10^6 HEK-293T cells were seeded in each well of a 6-well plate and grown overnight to 70–80% confluence. 2 μg of each plasmid (pMD2G, psPAX2, and CSII-EF-hB2M-A11) was mixed with Lipofectamine 3000 (Invitrogen, L3000015) for 20 min. The DNA-liposome mixture

was gently added to HEK-293T cells in the 6-well plate. After 6–8 h, the culture medium was replaced with 2 mL fresh medium per well. 48 h later, the hB2M-A11 lentiviral supernatant was harvested for transduction of T2-B2M^{KO}. 2 mL single cell suspension of 1 × 10^6 T2-B2M^{KO} was mixed with 2 mL lentiviral supernatant (1:1 dilution) plus 4 μg/mL protamine sulfate in a 6-well plate, and incubated at 37°C overnight. The HLA-ABC positive cells were sorted by FCM to obtain pure T2/A11 cell line (Supplemental Figure 6A). The HLA-A11 negative cells (SGC7901, BGC823, MB231, BT549 and PC9) were modified with lentiviral HLA-A*11:01 expression vector to obtain A11-positive cells and called with abbreviated name: SGC7901/A11, BGC823/A11, MB231/A11, BT549/A11, PC9/A11 in figures.

IFN-γ ELISPOT assay.

The number of peptide-reactive T cells was determined using ELISPOT assay. Briefly, T cell samples were collected after the completion of DC plus LP stimulation. T cells (1 × 10^5/well) were restimulated in vitro by 20h co-culture with LP-loaded DCs (1 × 10^4/wel) in RPMI 1640 medium in triplicate. Negative control included T cells and DCs in medium alone without peptide. Phytohaemagglutinin (PHA) (10μg/well) was added as positive control. The ready-to-use IFN-γ ELISPOT kit (Dakewe Biotech, Shenzhen, China) was adopted for the assay according to the manufacture's instruction. Plates were washed three times and incubated with a biotinylated anti-IFN-γ antibody. IFN-γ positive spots were developed using streptavidin–horseradish peroxidase (HRP) complex and diaminobenzidine (DAB) substrate. The number of IFN-γ spot-forming units (SFUs) present in each well was counted by C.T.L. Immuno Spot S6 Analyzer and

analyzed by Immuno Spot v6.0 software with the recommended parameters.

IFN-γ enzyme-linked immunosorbent assay.

Effector cells (1.0×10^5 TCR-transduced T cells) were co-incubated with target cells (1.0×10^5 T2/A11 or DC) loaded with 9-mer or 10-mer peptides (5 μg/mL) overnight in RPMI supplemented with 10% AB serum and 50 IU/mL IL2 in 96-well flat-bottom plates. The supernatants were collected to measure the concentration of IFN-γ by enzyme-linked immunosorbent assay (ELISA) (Dakewe Biotech) following manufacturer's instructions. In the assay of tumor cell recognition, 2×10^5 TCR-T cells or control cells were co-cultured with 1×10^5 tumor cells (SGC7901, SGC7901/A11, SGC7901/CT83^{KO}, BGC823/A11, MB231/A11, BT549/A11, HCC827, PC9/A11) for 24h, and supernatants were collected and analyzed the IFN-γ by ELISA.

CTA bioinformatics analysis.

To identify the optimal target antigens of CTAs, we obtained 224 CTA genes from GenBank and searched the GTEx Portal database (https://www.gtexportal.org/home/index.html) to analyze their expressions with strict criteria for specific expression in testis: 1. Strictly no expression in vital organs/tissues including brain and heart: protein-transcript per million (pTPM) ≤0.1; 2. Slight expression in not more than one non-vital tissue type except testis: pTPM >1.0 ~ < 3.0. Next, we searched TCGA database to rank positive rates and mRNA expression levels of the eligible CTAs in the commonest solid tumors including LUAD, LUSC, STAD and BRCA. To excavate TAA expression on thymus, we collected the data of three thymus sample 1599, 1602 and 1603 from HPA database as well as from FANTOM5 CAGE data of 0.5, 0.5, 0.83

years old infant, male. Here CTAs contains the 92 cancer/testis genes screened in Supplemental Table 4; tumor differentiation antigens (TDAs) contains 17 genes including PMEL (GP100), MLANA (MART-1), CEACAM1, CEACAM3, CEACAM4, CEACAM5 (CEA), CEACAM6, CEACAM7, CEACAM8, RAB38 (NY-MEL-1), ANKRD30A (NY-BR-1), GPR143 (OA1), ACP3 (PAP), KLK3 (PSA), TYRP1 (TRP1), DCT (TRP2), TYR (Tyrosinase); and tumor overexpressed antigens (TOAs) contains 19 genes including AFP, CALCA, EZH2, GPC3, HPN (Hepsin), ERBB2 (HER2), EGFR, TP53, BIRC5 (Survivin), KLK4 (Kallikrein-4), KIF20A, MDK (Midkine), MUC1, PRAME, FOLH1 (PSMA), MOK (RAGE-1), STEAP1, TERT (Telomerase), WT1. For HPA data, nTPM (normalized transcripts per million) values give a quantification of the gene abundance which is comparable between different genes and samples; for FANTOM5 CAGE data, RNA expression in human tissues obtained through Cap Analysis of Gene Expression (CAGE) generated by the FANTOM5 project are reported as Scaled Tags Per Million.

Cross-reactive epitope analysis.

After inferring the essential non-anchor residues in the verified epitope, the potential cross-reactive peptides of TCR1 were analyzed by the online tool Expitope2.0 (http://webclu.bio.wzw.tum.de/expitope2/). The peptides in human proteins were analyzed for the mismatches compared to the verified epitope and predicted for the binding affinity with HLA-A*11:01.

Supplementary figures

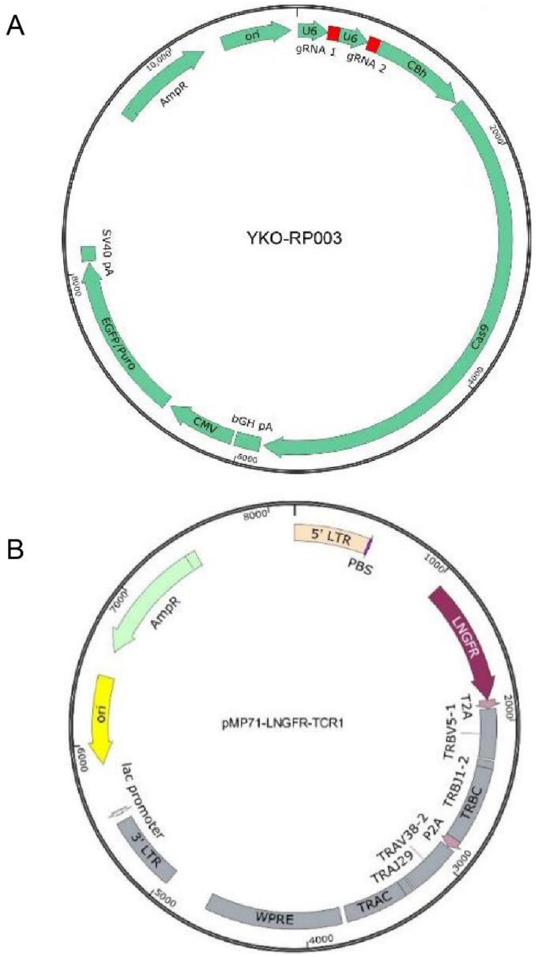

Supplemental Figure 1. The structure diagram of plasmids. (A) The CT83 KO plasmid: two CT83 sgRNAs, Cas9 coding sequence, the EGFP and Puro genes are included. (B) CT83/TCR1 retroviral vector. The paired TCR1 ab V genes were respectively linked with mouse TCR ab C genes and cloned into pMP71 retroviral vector. A P2A cleavage sequence was added between the TCR chains, and a T2A-linked delta-LNGFR (CD271) gene was included as a biomarker for monitoring of TCR expression.

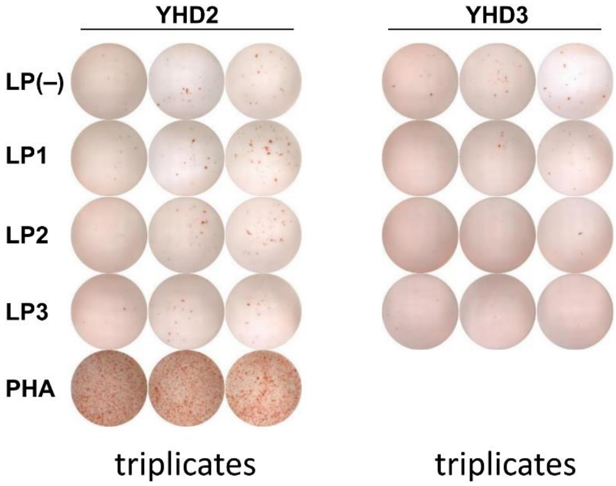

Supplemental Figure 2. Stimulation of CT83-reactive T cells from YHD2 (HLA-B*46:01+) and YHD3 (HLA-C*01:02+). PBMCs were stimulated by co-cultured with CT83 LP1-3 loaded autologous DCs for three rounds in vitro and analyzed by IFN-γ ELISPOT assay. No peptide (LP-) wells was used as negative control, while PHA wells was added as the positive control for both YHD2 and YHD3.

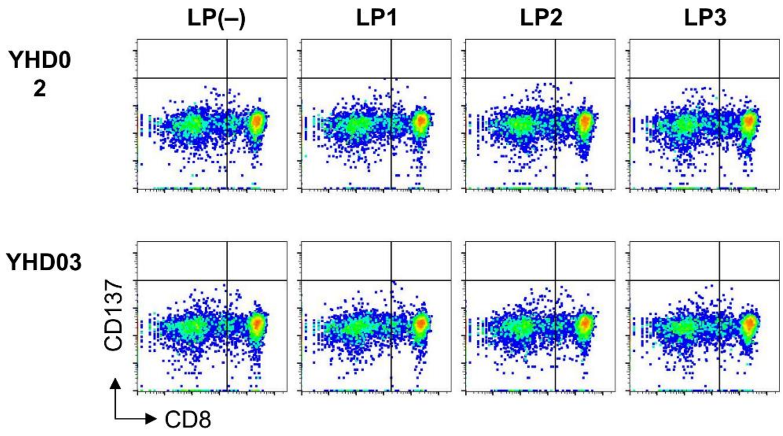

Supplemental Figure 3. FCM analysis of CT83 LP-reactive T cells for YHD2 and YHD3. The CD137+ cell subpopulation was detected by antibody staining and defined as CT83 LP-reactive CD8+ T cells. No obvious difference was observed in the proportion of CD8+CD137+ T cells between the experimental groups and the control group, for both YHD2 and YHD3.

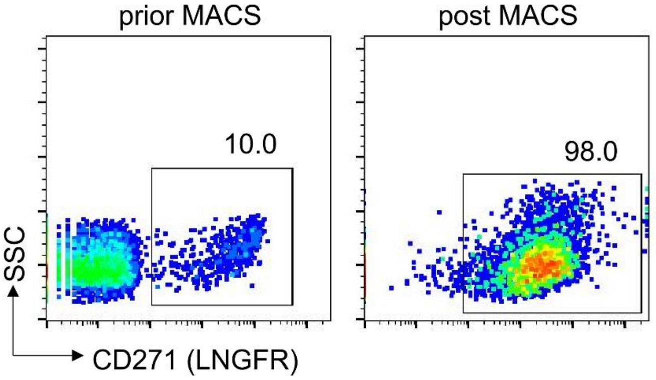

Supplemental Figure 4. Repesentative FCM data showing the enrichment of TCR1-T cells. CD271 was co-expressed and used as a marker for the detection of the transduced cells with FCM. The enrichment was performed using magnetic activated cell sorting (MACS). Left panel: TCR-Ts before MACS; right panel: after MACS.

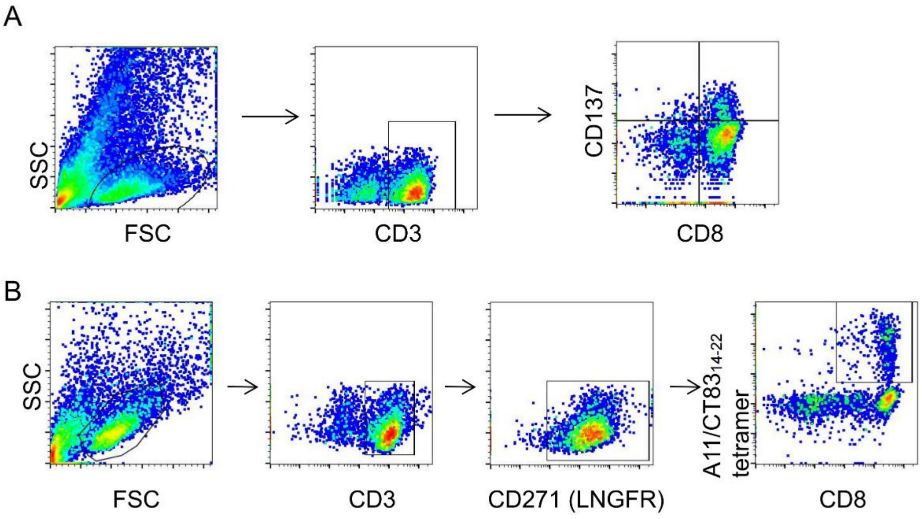

Supplemental Figure 5. FCM gating strategy used in analysis of the CT83-reactive T cells staining with CD3, CD8 and CD137 antibodies. (A) The CT83 LP-stimulated or TCR1-T cells staining with CD3, CD271 antibodies and gating with CD3. (B) TCR-T cells staining with CD3, CD271 antibodies and A11/CT8314-22 tetramer, gating with CD3 and CD271 for A11/CT8314-22 tetramer cells.

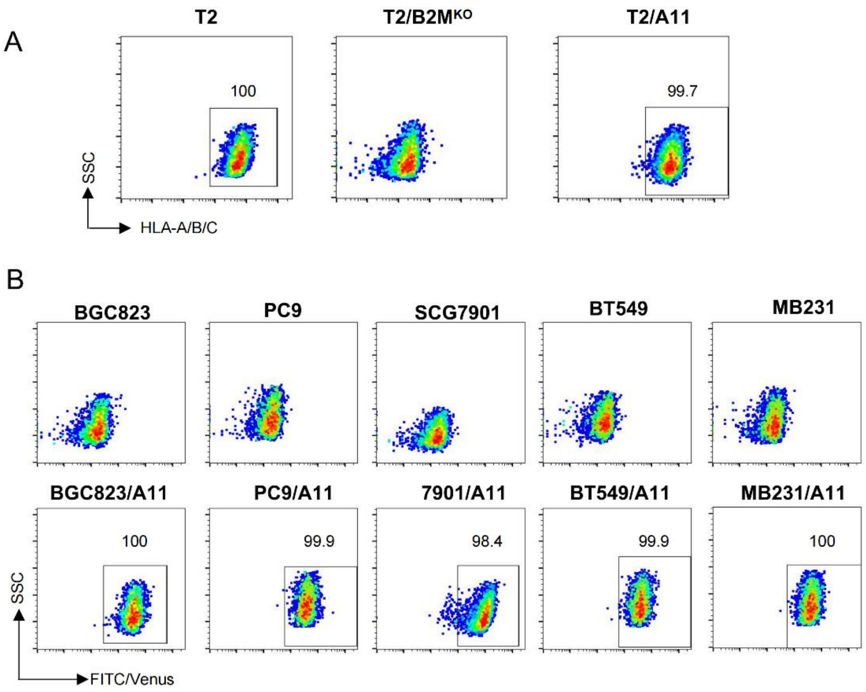

Supplemental Figure 6. FCM analysis of HLA-A*11:01-engineered cell lines. (A) The endogenous HLA expression of T2 cells was first removed via B2M KO by CRISPR-Cas9 targeting, and HLA-A*11:01 expression was then achieved by transduction of A11 vector to establish T2/A11 cell line. The original, B2M KO (T2/B2MKO) and HLA-A*11:01 transduced T2 (T2/A11) cells were stained with anti-HLA-ABC antibody. (B) The HLA-A*11:01 positive tumor cell lines (BGC823/A11, 7901/A11, BT549/A11, PC9/A11 and MB231/A11) were established by transduction with HLA-A*11:01 expression vector. Venus gene included in the HLA-A*11:01 expression vector was used as a biomarker for HLA-A11 expression.

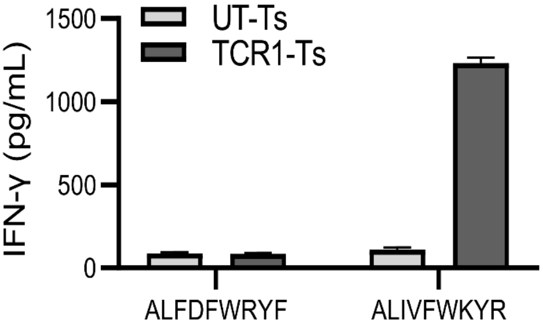

Supplemental Figure 7. Study of the off-target effect of TCR induced by homologous epitope By Expitope2.0 search for candidate peptides that shared at least 4/9 residues were tested for CT83/TCR1-T recognition

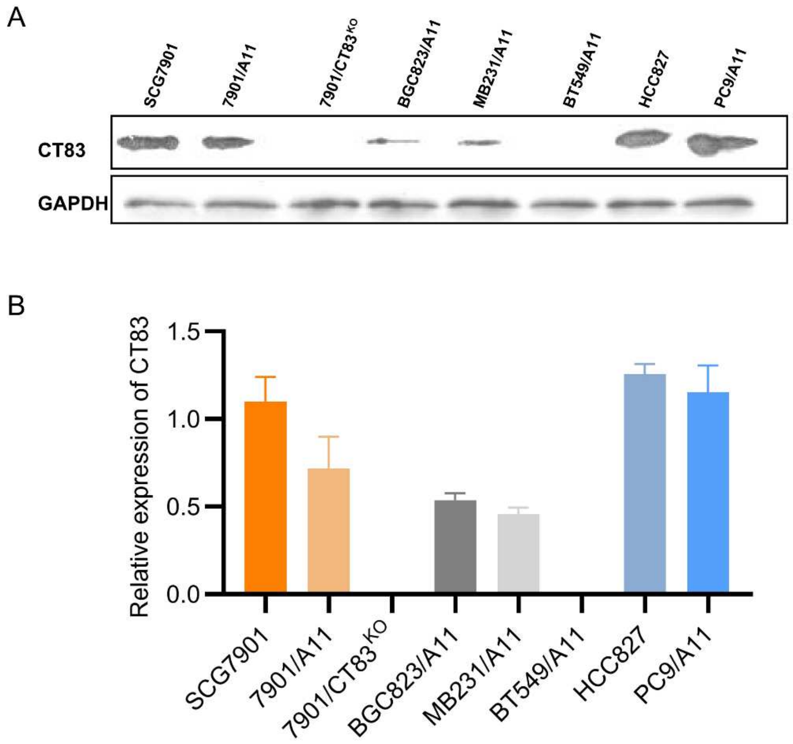

Supplemental Figure 8. CT83 expression in eight tumor cell lines including both primary and HLA-A*11:01 engineered lines. (A) Representative photo of western blotting with CT83 antibody staining for blotting membrane. GAPDH was used as the loading control. (B) Graph of the statistical analysis of WB. The relative amounts of proteins were normalized to GAPDH. Tumor cell lines were derived from stomach cancer (SGC7901 and BGC823), breast cancer (MD231 and BT549) and lung cancer (HCC827 and PC9). Data are means ± SD, n=3 replicates.

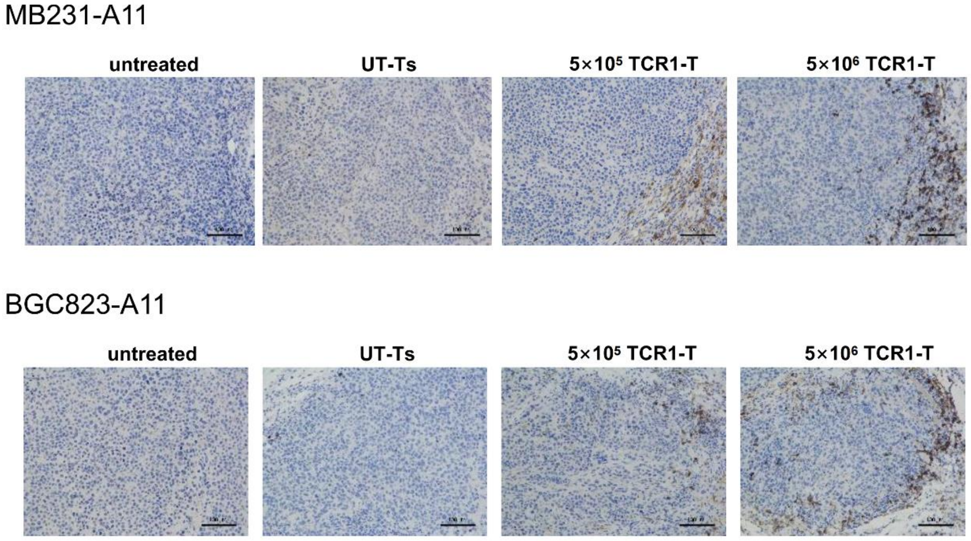

Supplemental Figure 9. T cell marker CD3 IHC staining of T cell infiltration in tumor sections from MB231/A11 and BGC823/A11 cell lines. Note: untreated (without injection with T cells) and UT-Ts (injected with untransduced T cells) were used as control groups. The experimental groups were injected with different doses of TCR1-T cells (5×105and 5×106 TCR1-T cells respectively). Scale bars: 100 μm.

Supplemental Table 1. Prediction of the CT83 peptide bindings to HLA-I molecules

pos	HLA	peptide	Affinity(nM)	%Rank	BindLevel
4	HLA-C*01:02	YLLLASSIL	2522.8	0.2279	<=SB
6	HLA-C*01:02	LLASSILCAL	9059.6	1.6342	<=WB
7	HLA-B*46:01	LASSILCAL	6140.34	0.7	<=WB
7	HLA-C*01:02	LASSILCAL	2779.3	0.267	<=SB
9	HLA-B*46:01	SSILCALIVF	9835.35	1.4	<=WB
10	HLA-A*11:01	SILCALIVFWK	110.11	0.7	<=WB
10	HLA-B*46:01	SILCALIVF	7080.88	0.9	<=WB
11	HLA-A*11:01	ILCALIVFWK	48.26	0.4	<=SB
13	HLA-A*11:01	CALIVFWKYR	596.37	2	<=WB
14	HLA-A*11:01	ALIVFWKYRR	298.56	1.4	<=WB
14	HLA-A*11:01	ALIVFWKYR	397.24	1.6	<=WB
16	HLA-C*01:02	IVFWKYRRF	9403.8	1.7307	<=WB
23	HLA-B*46:01	RFQRNTGEM	11645.16	1.9	<=WB
24	HLA-B*46:01	FQRNTGEM	5148.09	0.6	<=WB
23	HLA-C*01:02	RFQRNTGEM	8407.1	1.4542	<=WB
30	HLA-C*01:02	EMSSNSTAL	7563.9	1.2372	<=WB
31	HLA-C*01:02	MSSNSTALAL	6289.1	0.946	<=WB
31	HLA-C*01:02	MSSNSTAL	8122.7	1.3709	<=WB
32	HLA-C*01:02	SSNSTALAL	1430	0.0816	<=SB
40	HLA-C*01:02	LVRPSSSGL	3507.5	0.3847	<=SB
52	HLA-B*46:01	NTDNNLAVY	9779.22	1.4	<=WB
58	HLA-C*01:02	AVYDLSRDI	9137	1.6566	<=WB
60	HLA-B*46:01	YDLSRDILNNF	5877.27	0.7	<=WB
59	HLA-C*01:02	VYDLSRDIL	6557.9	1.0075	<=WB
62	HLA-B*46:01	LSRDILNNF	2674.43	0.3	<=SB
66	HLA-C*01:02	ILNNFPHSI	3366.6	0.3617	<=SB
74	HLA-C*01:02	IARQKRILV	7006	1.0964	<=WB
81	HLA-A*11:01	LVNLSMVENK	99.24	0.7	<=WB
85	HLA-B*46:01	SMVENKLVEL	11105.69	1.8	<=WB
85	HLA-C*01:02	SMVENKLVEL	9619.2	1.7873	<=WB
86	HLA-C*01:02	MVENKLVEL	2613	0.2421	<=SB
90	HLA-C*01:02	KLVELEHTL	7047.6	1.1061	<=WB
92	HLA-A*11:01	VELEHTLLSK	590.09	2	<=WB
91	HLA-C*01:02	LVELEHTLL	6943.5	1.0844	<=WB
101	HLA-B*46:01	KGFRGASPH	9688.86	1.4	<=WB

NetMHC 4.0 was adopted for HLA-A*11:01 and HLA-B*46:01, while NetMHCpan 4.0 was used for HLA-C*01:02.

Supplemental Table 2. The HLA-I genotyping of health donors' PBMCs and tumor cell lines

	A		B		C	
YHD1	A*11:01	A*11:01	B*55:02	B*40:01	C*03:03	C*08:01
YHD2	A*26:01	A*24:02	B*55:02	B*46:01	C*03:03	B*58:01
YHD3	A*02:07	A*33:03	B*15:01	B*15:02	C*01:02	C*03:04
SCG7901	A*68:02	A*68:02	B*15:03	B*15:03	C*12:03	C*12:03
BG823	A*68:02	A*68:02	B*15:03	B*15:03	C*12:03	C*12:03
MB231	A*02:01	A*02:01	B*41:01	B*40:02	C*02:02	C*04:01
BT549	A*23:01	A*30:02	B*53:01	B*27:03	C*12:03	C*12:03
HCC827	A*11:01	A*11:01	B*52:01	B*52:01	C*12:02	C*12:02
PC9	A*02:06	A*24:02	B*39:01	B*55:02	C*07:02	C*03:03

Supplemental Table 3. Top 10 clones in scTCR sequencing using 10x Genomics platform.

clonotype	chain	v_gene	j_gene	c_gene	proportion
1	TRA	TRAV38-2	TRAJ29	TRAC	0.20801
2	TRA	TRAV21	TRAJ33	TRAC	0.031245
3	TRA	TRAV20	TRAJ49	TRAC	0.020542
4	TRA	TRAV29	TRAJ33	TRAC	0.020024
5	TRA	TRAV27	TRAJ23	TRAC	0.013292
6	TRA	TRAV26-1	TRAJ56	TRAC	0.012774
7	TRA	TRAV13-2	TRAJ29	TRAC	0.008631
8	TRA	TRAV41	TRAJ43	TRAC	0.008458
9	TRA	TRAV36	TRAJ40	TRAC	0.008458
10	TRA	TRAV16	TRAJ34	TRAC	0.004316
1	TRB	TRBV5-1	TRBJ1-2	TRBC1	0.20801
2	TRB	TRBV6-2	TRBJ2-1	TRBC1	0.031245
3	TRB	TRBV6-6	TRBJ2-3	TRBC2	0.020542
4	TRB	TRBV30	TRBJ1-5	TRBC1	0.020024
5	TRB	TRBV29-1	TRBJ1-2	TRBC1	0.013292
6	TRB	TRBV6-5	TRBJ2-2	TRBC2	0.012774
7	TRB	TRBV28	TRBJ1-1	TRBC1	0.008631
8	TRB	TRBV6-6	TRBJ2-1	TRBC1	0.008458
9	TRB	TRBV4-1	TRBJ2-7	TRBC2	0.007423
10	TRB	TRBV13	TRBJ2-7	TRBC2	0.004316
